# Review Update on the Life Cycle, Plant–Microbe Interaction, Genomics, Detection and Control Strategies of the Oil Palm Pathogen *Ganoderma boninense*

**DOI:** 10.3390/biology11020251

**Published:** 2022-02-06

**Authors:** Izwan Bharudin, Anis Farhan Fatimi Ab Wahab, Muhammad Asyraff Abd Samad, Ng Xin Yie, Madihah Ahmad Zairun, Farah Diba Abu Bakar, Abdul Munir Abdul Murad

**Affiliations:** 1 Department of Biological Sciences and Biotechnology, Faculty of Science and Technology, Universiti Kebangsaan Malaysia, UKM, Bangi 43600, Malaysia; anis.aw@fgvholdings.com (A.F.F.A.W.); P99743@siswa.ukm.edu.my (M.A.A.S.); P108611@siswa.ukm.edu.my (N.X.Y.); madihah@mpob.gov.my (M.A.Z.); fabyff@ukm.edu.my (F.D.A.B.); munir@ukm.edu.my (A.M.A.M.); 2 Fraser’s Hill Research Centre (PPBF), Faculty of Science & Technology, Universiti Kebangsaan Malaysia, UKM, Bangi 43600, Malaysia; 3 FGV Innovation Centre (Biotechnology), Pt. 23417 Lengkuk Teknologi, Bandar Enstek 71760, Malaysia; 4 Plant Pathology & Biosecurity Unit, Biology & Sustainability Research Division, 6, Malaysian Palm Oil Board, Bandar Baru Bangi, Kajang 43000, Malaysia

**Keywords:** basal stem rot, biological control, *Ganoderma*, fungal pathogen, mating, palm oil, plant–microbe interactions

## Abstract

**Simple Summary:**

Palm oil is one of the main crops produced in Southeast Asia. However, the palm oil plantations within the region were severely affected by the basal stem rot (BSR) disease, caused by *Ganoderma boninense*. The disease causes substantial economic losses to oil palm producers, especially Indonesia and Malaysia. This review will cover the current knowledge on *G. boninense* published within the last 10 years.

**Abstract:**

Plant pathogens are key threats to agriculture and global food security, causing various crop diseases that lead to massive economic losses. Palm oil is a commodity export of economic importance in Southeast Asia, especially in Malaysia and Indonesia. However, the sustainability of oil palm plantations and production is threatened by basal stem rot (BSR), a devastating disease predominantly caused by the fungus *Ganoderma boninense* Pat. In Malaysia, infected trees have been reported in nearly 60% of plantation areas, and economic losses are estimated to reach up to ~USD500 million a year. This review covers the current knowledge of the mechanisms utilized by *G. boninense* during infection and the methods used in the disease management to reduce BSR, including cultural practices, chemical treatments and antagonistic microorganism manipulations. Newer developments arising from multi-omics technologies such as whole-genome sequencing (WGS) and RNA sequencing (RNA-Seq) are also reviewed. Future directions are proposed to increase the understanding of *G. boninense* invasion mechanisms against oil palm. It is hoped that this review can contribute towards an improved disease management and a sustainable oil palm production in this region.

## 1. Introduction

Oil palm is one of the most important oil-producing crops in the world, as it contributes ~34% of the world’s vegetable oil and fat supply [1]. However, the sustainability of oil palm plantations and palm oil production in Southeast Asia is affected by pests such as the bagworm and the beetles *Rhynchophorus ferrugineus* and *Oryctes rhinoceros* [2,3,4], as well as diseases like upper stem rot (USR) and basal stem rot (BSR), caused by fungi of the genus *Ganoderma* [5,6,7]. BSR has by far been the most serious disease of oil palm in Malaysia in the last 30 years, with increasing disease incidents and infection rates from 1.5% in 1995 to 7.4% in 2017 [8]. Typically, plants infected by the fungus stop producing fruits and die within 2–3 years [9,10]. Infected oil palms usually produce lower yields due to the reduced weight of fruit bunches. Economic losses due to stem rot diseases in Malaysia are estimated to reach values of up to USD500 million a year [11].

Multiple species of *Ganoderma* have been reported to incite BSR disease in oil palm in Southeast Asia, namely *Ganoderma boninense*, *Ganoderma zonatum* and *Ganoderma miniatocinctum* [12]. Amongst these species, *G. boninense* is the most prominent causal agent of both BSR and USR [13,14]. By 2020, the total plantation area affected by BSR is estimated to be around 443,430 ha (equivalent to 65.6 million oil palms). Together, both BSR and USR are the most severe diseases affecting oil palm plantations across Southeast Asia [15], reducing yield, increasing plant mortality and necessitating replanting [11].

**Figure 1:**
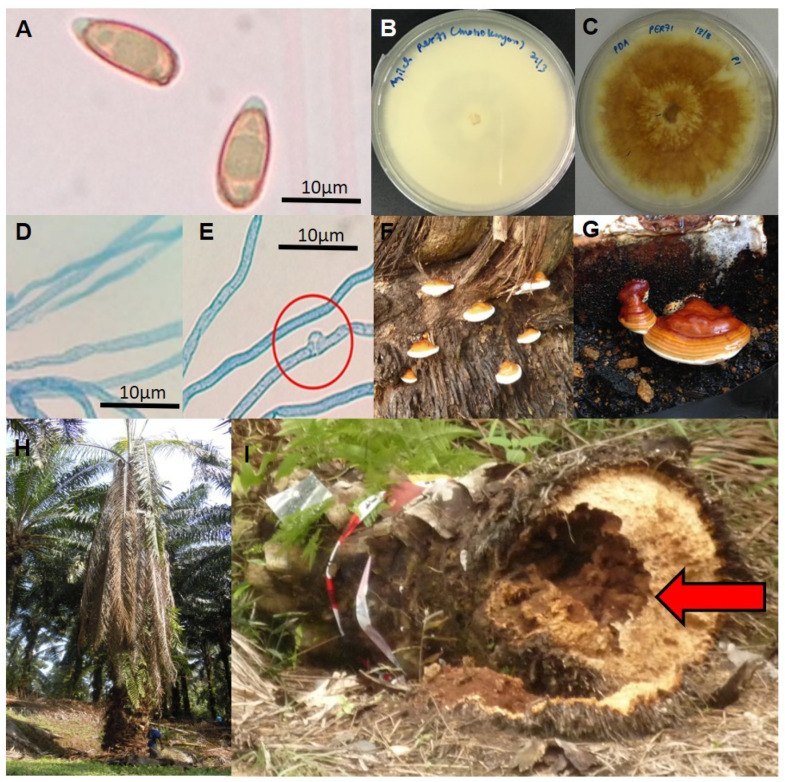
The morphological diversity of *G. boninense* observed during different life stages. (**A**) Basidiospore structure observed under the light microscope (100X magnification); (**B**) Monokaryotic mycelia of *G. boninense* PER71 grown on Potato Dextrose Agar (PDA) (age 7 days); (**C**) Dikaryotic mycelia of *G. boninense* PER71 grown on PDA (age 7 days); (**D**) Monokaryotic mycelia observed under the light microscope (100X magnification); (**E**) Dikaryotic mycelia observed under the light microscope (100X magnification) with the presence of a clamp connection in dikaryotic mycelia (red circle); (**F**) Formation of basidiocarps on the basal stem of the infected oil palm tree; (**G**) Formation of basidiocarps on rubber wood block (artificial inoculation and formation of *G. boninense* basidiocarps); (**H**) The symptoms of BSR disease observed on oil palm trees, such as lower leaves collapsing and hanging downwards vertically from the point of attachment to the trunk; (**I**) The oil palm trunk falls over due to the decay of the inside basal stem (red arrow). Scale bars: 10 μm (**A**,**D**,**E**).

*Ganoderma* is a white-rot fungus that belongs to the family *Ganodermataceae* and the class *Agaricomycetes*. As with all members of *Basidiomycota*, *Ganoderma* have specialized reproductive spores (or basidiospores) that are important in maintaining the sexual cycle and also serve as the main air dispersal unit. *Ganoderma boninense* is characterized by large and woody bracket basidiocarps, which typically grow on the trunks of trees and, like all other white-rot fungi, degrade the lignin component in wood (Figure 1).

## 2. *G. boninense* Life Cycle

**Figure 2:**
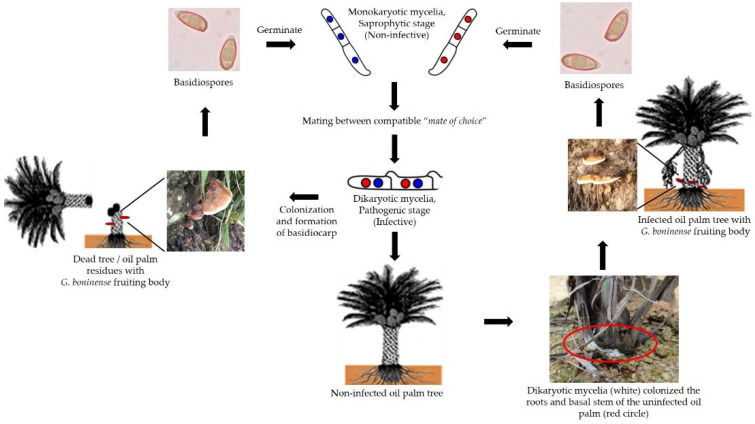
The life cycle of *G. boninense*. The *G. boninense* fruiting bodies produce millions of basidiospores which can be spread by numerous vectors, such as wind and animals. The basidiospore germinates to form mycelia (monokaryotic form), which is non-pathogenic to the oil palm tree [33]. The mating between compatible mates of choice (monokaryon/dikaryon) will form the pathogenic dikaryotic mycelia. The dikaryotic mycelia then begins to colonize the root and basal stem of the oil palm tree. Dikaryotic mycelia will undergo hyphae morphogenesis to form a needle-like structure to facilitate the penetration into the host cells. *G. boninense* infection of the oil palm tree will manifest as the formation of basidiocarps on the basal stem of the infected tree. However, there are cases where the palms dies and collapses in the field without the formation of a *G. boninense* fruiting body.

Basidiomycetes employ two different strategies for reproduction: sexual spores and vegetative mycelia. Although *G. boninense* possesses both mechanisms of reproduction, basidiospores are believed to be the primary source of inoculum [16], as they are easily dispersed by wind or animal vectors. Once it reaches a suitable environment, the basidiospores germinate to form monokaryotic vegetative mycelia. The monokaryotic mycelia typically grow saprophytically in the environment by feeding on dead plant material. Nuclear exchange and migration follow, resulting in the formation of a dikaryotic mycelium, which then invades and establishes itself within the plant host. The dikaryotic mycelia later gives rise to the formation of the fruiting body (Figure 1F) under appropriate environmental conditions. The fruiting body (or basidiocarp) is a multicellular reproductive structure in which karyogamy occurs and meiotic spores are produced. The sexual cycle of *Ganoderma* sp. is completed once the basidiocarp produces basidiospores (Figure 2). The regulation of sexual reproduction by the tetrapolar mating system promotes outbreeding and the diversity of their genetic content in the same plantation area, thus resulting in dynamic populations, and is most probably the primary cause that leads to inefficient disease management [16,17]. The genetic variability was reported to be higher especially for isolates from different geographical origins. Due to the high divergence of the genetic pool between distinct isolates, different strains exhibit a different degree of aggressiveness and tolerance toward biological control and fungicides [6,17,18].

Previous reports have suggested that *G. boninense* is a heterothallic species with bifactorial incompatibility due to their possession of two unlinked mating-type loci, known as mating-type A (matA) and mating-type B (matB) [19,20]. Further studies showed that this species has multiple alleles at both mating-type loci, with at least 81 matA and 83 matB alleles [21]. Studies have also shown that *G. boninense* requires a compatible partner in order to mate and initiate the sexual cycle—a common feature of heterothallism. Only monokaryons possessing two unlinked mating-type loci are able to initiate the mating pathway and undergo the complete sexual cycle, as ruled by the tetrapolar mating system [18]. The first locus, usually known as matA, is biallelic and harbors genes encoding for homeodomain transcription factors, while the second locus, known as matB, is multiallelic and contains pheromone receptor genes and pheromone precursors [22]. Our initial analyses using the available genomic data indicated that the matA locus of *G. boninense* covers about 360 kilobases in size, and includes genes encoding for both homeodomain transcription factors 1 and 2 (unpublished data), which is similar to those reported in other *Ganoderma* sp. [23]. In addition, the matB locus of *G. boninense* contains at least ten pheromone receptors and four pheromone precursors (unpublished data). The *matB* genes also play a critical role in mate recognition and choreographs the interactions between prospective mating partners. The initial stage of mating after hyphal fusion begins through the activation of the pheromone response pathway. The activation of the mating pathway is also necessary to override the heterokaryon incompatibility system that is ordinarily triggered when different and genetically distinct hyphae fuse [24]. Other than mate recognition, the *matB* genes are also essential for nuclear migration, septal dissolution and clamp cell fusion [25].

The binding of the pheromones with compatible pheromone receptors activates the transcription of genes related to the mating process through the MAP kinase-mediated signaling cascade [26]. The successful activation of the pheromone response pathway leads to the dissolution of septa and plasmogamy. Thereafter, the *matA* genes homeodomain 1 (HD1) and homeodomain 2 (HD2) will serve as the second incompatibility checkpoint. Compatible mating partners bring together the HD1 and HD2 that heterodimerize by polar-hydrophobic interaction, thereby generating an active transcription factor complex that commits mated cells to sexual development [27]. The *matA* genes are essential for nuclear pairing, clamp cell formation and coordinating nuclear division and are also involved in clamp cell septation. Both *matA* and *matB* genes work coordinately to maintain the successfully established dikaryotic stage. In *Ustilago maydis*, mating-type genes are also categorized as virulence factors because they are involved in the activation of other pathogenesis-related genes [28]. Besides, mating is also crucial in ensuring survival and maintaining the genetic variation of many pathogenic fungi.

The mating event provides numerous advantages: it gives rise to novel gene combinations and facilitates the adaptation of the species toward changing environments; it serves to remove mutations that have arisen within the genome as well as increase the efficiency of DNA repair via homologous recombination, in which the second copy of the nucleus will act as a template for DNA repair; and it helps raise the possibility of the fungi establishing better-suited structures and mechanisms for host invasion [29,30], as has been shown in other basidiomycete fungal pathogens, such as *Puccinia graminis* f. sp. *tritici* [31] and *U. maydis* [32].

## 3. Plant–Microbe Interaction

The mechanism of *G. boninense* infection of oil palm and its dispersal within oil palm plantations is not fully understood. An earlier study suggested that the colonization of *Ganoderma* sp. in the oil palm field is achieved through several methods, that include contact between healthy and diseased roots [34]. Colonization by mycelia can also occur on wounded or dead roots. As the oil palm roots can continue to grow beyond four planting rows, this inevitably results in root-to-root contact between palms, enabling the spread of *Ganoderma* sp. The growing presence of patches due to BSR infection over time has also led to the theory that roots are the primary source of inoculum in the field [35]. Furthermore, oil palm debris that has been colonized by *G. boninense* mycelia can also act as an inoculum source, thus sustaining the spread of *G. boninense* even after the replanting of new trees [33].

Management of BSR is difficult and challenging, as the formation of infective dikaryotic mycelia continues through mating between the mycelia and newly germinated basidiospores. The basidiospores can travel long distances through wind and disseminate in the same, or even across different plantation areas [16,21]. The fungus then grows along and invades the roots through wounds or by puncturing the healthy plant cells [36] through the formation of needle-like structures [37] that eventually penetrate the epidermis layer of the oil palm roots. At advanced stages of infection, the fungal hyphae can be detected in the xylem, phloem, pith and parenchymal cells [36,38,39]. The formation of black lines within the infected tissues will lead to the infection in the stem [40]. Under microscopic observation, the black lines are seen as embedded thick-walled, swollen structures of the *G. boninense* hyphae, which are postulated to play a crucial role in long-term survival in the soil [40] by generating a resistant barrier against other soil microorganisms. Conversely, monokaryotic mycelia remain non-pathogenic due to their incapability of causing damage to—and infecting—the oil palm [41,42].

Being a hemibiotroph, *G. boninense* is able to switch their lifestyles between biotrophic and necrotrophic phases, depending on the environment and its conditions [36,43]. The fungus exhibits a biotrophic phase during initial stages of infection when hyphae colonize the host plant. The transition to the necrotrophic phase, which involves extensive cell wall degradation, occurs once the pathogen overcomes the host defenses [11,44]. Other pathogenic fungi with similar modes of infection include *Botrytis cinerea*, *Rhizoctonia solani* and *Sclerotinia sclerotiorum* [44,45]. These pathogens produce small cell wall-degrading enzymes (CWDEs), including cellulase, laccase, polygalacturonase and manganese peroxidase, to soften and loosen the host cell wall without detrimentally affecting the host cells [46]. During early infection of the oil palm root, *G. boninense* hyphae will colonize the roots and secrete trace amounts of CWDEs, including polygalacturonase and laccase [47,48], to enable the establishment of a continued supply of nutrients from the living cells of their hosts. The secreted CWDEs will then degrade the host cell wall and affect the integrity of the cell-wall polysaccharides by triggering the release of damage-associated molecular patterns (DAMP) molecules by the host [47,49]. These endogenous danger molecules interact with transmembrane pattern recognition receptors to activate the plant host’s primary innate defense response, also known as pathogen-associated molecular pattern-triggered immunity (PTI) [50]. This process initiates the production of several response molecules such as reactive oxygen species (ROS) and phytoalexins, and trigger cell wall alterations and the accumulation of pathogenesis-related (PR) proteins [51,52].

Furthermore, the oil palm also initiates a secondary defense system, known as effector-triggered immunity (ETI), to monitor the presence of effectors secreted by the pathogen [52]. The presence of an effector triggers a hypersensitive response (HR) that will execute programmed cell death (PCD) and other locally induced defense responses to restrict the growth of *G. boninense* [43]. The increased plant defense responses during the biotrophic phase will trigger ROS overproduction, causing cellular damage in the fungus and a change toward the necrotrophic lifestyle [43]. A phytopathogen’s biotrophic phase can often be prolonged due to evolution under selection pressure in order to avoid host defense responses and weaken the ETI of the host [44]. Effectors are important to suppress PTI and HR, assisting disease development by inducing the abscisic acid (ABA) pathway and producing metabolites to suppress jasmonic acid (JA)- and salicylic acid (SA)-induced defense responses by the plant host [44,52,53]. The failure of the plant host to detect the effectors will reduce the production of plant resistance proteins (RPs), whereas the failure to trigger ETI leads to the susceptibility of the pathogen. During the necrotrophic phase, the fungus will secrete various phytotoxic compounds and CWDEs to cause nutrient leakage [43,52]. As the infection becomes more severe, hyphae outside the root will form tough and melanized mycelia by encapsulating the thin-walled hyphae with layers of thick-walled cells around the roots, resulting in massive hyphae aggregations and the formation of basidiocarps [36]. The presence of basidiocarps on an infected palm signifies that the plant is in the severe infection stage, and the progression of decay has occurred inside the plant. Over time, the plant will die, and the necrotrophic phytopathogen causing plant death will continue to live saprotrophically.

The microscopic examination of infected palms at the cellular level revealed the establishment of biotrophic nutrition by *G. boninense* during colonization. The fungus degrades the lignin to weaken the rigid plant cell-wall structure prior to starch consumption [36]. Generally, three key events occur during host colonization by hemibiotrophs, namely, (i) penetration, (ii) nutrient absorption at the expense of the host, and (iii) host death [36]. Currently, little is known on the proteins or enzymes involved during host penetration, whereas the second and third components are well characterized in *G. boninense* pathogenesis. A recent study has shown that a reduction of *G. boninense* colonization occurred along with a significant up-regulation of the pathogenesis-related protein 1-like (PR-1) gene, which is produced as part of the oil palm’s defense mechanism [43]. They further proposed that the high expression of PR-1 resulted in the sequestration of ergosterol, a part of the primary metabolite and cell-wall component in *G. boninense*. PR-1 protein has been reported to have fungicidal properties and is produced by the host plant to combat the further invasion of the host by fungal pathogens [43].

**Figure 3:**
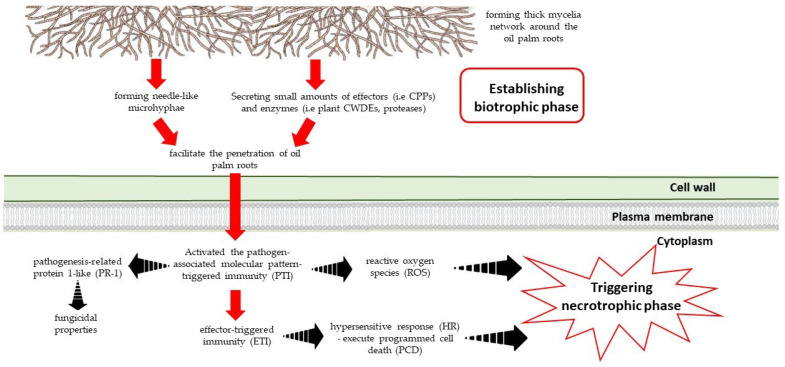
The interaction between fungal pathogen (*G. boninense*) and the plant (oil palm root).

Recently, it was revealed that *G. boninense* colonizes the host by forming needle-like microhyphae that facilitate the penetration of oil palm roots [37]. The thin-walled microhyphae usually have an extracellular matrix that undergoes a highly localized degradation of its cellulose component, resulting in a matrix that extends into minute cracks of the cell wall (Figure 3). This feature has also been observed in other fungal pathogens such as *Fusarium oxysporum* [54] and *Heterobasidion parviporum* [55]. In contrast, the microhyphae produced by *Neurospora crassa*, *Ophiostoma ulmi* and *Phellinus noxius* are activated during growth in the presence of inhibitors [56]. In response, the plant reacts by enhancing ROS production through an oxidative burst [57].

## 4. *G. boninense* Multi-Omics Data

With the advancement and improvement of sequencing technologies, genome and transcriptome sequencing has become the approached method in understanding microbial interactions with the environment [58,59], animal hosts [60] and plant hosts [61,62]. Currently, the genomes of five pathogenic strains of *G. boninense* have been sequenced: two strains isolated from Indonesia, i.e., the strains NJ3 and G3 [63,64]; and three strains isolated from Malaysia, i.e., PER71, BRIUMSc [65] and FGV-M [66]. All genome datasets are freely available from the NCBI database (Table 1).

The difference in the genome size between all the sequenced *G. boninense* strains suggests that a high genome diversity exists between these strains, and perhaps, portions of the genome have suffered duplications or deletions during the evolution of the various strains. In addition, the complete mitochondrial genome of this fungus is 86,549 bp. The total number of genes encoding for proteins is 51, with 15 conserved proteins, 4 hypothetical proteins, 5 homing endonucleases and 27 tRNAs, as well as small and large rRNA subunits [67]. Furthermore, a pan-genome analysis of eight draft genomes of *Ganoderma* sp. showed that of 35,121 orthologous genes (OGs), only 4898 genes are shared in all species [68]. The remaining 30,223 genes were classified as accessory genes in the genome, among which 1905 are species-specific genes. Interestingly, comparative genomic analyses between the non-pathogenic *G. lucidum* and the pathogenic *G. boninense* led to the discovery of 607 genes found only in the genome of *G. boninense*, thus suggesting their role in *G. boninense*‘s pathogenicity toward its host [68]. Most phytopathogenic fungi have well-established arsenals of carbohydrate-active enzymes (CAZymes), including CWDEs secreted during infection. A recent study has shown that the *G. boninense* genome has 755 CAZyme- encoding genes, of which 465 encode for CWDEs, including carbohydrate esterases (CE), glycoside hydrolases (GH) and polysaccharide lyases (PL) [47].

The genomic analysis of the publicly available genome databases of *Ganoderma* sp. strain 10,597 SS1, *G. lucidum* strain Xiangnong No.1, *G. lucidum* BCRC 37,177 and *G. lucidum* strain G.260125 has found that the products of the *matA* genes from all strains are highly dissimilar in sequence, and hence represent four different mating type specificities [23]. As for the *matB* genes, around 8 to 9 different pheromone receptor genes and 10 pheromone precursors were found to be located within a 60–100 kb long sequence region in all 4 genome databases.

Three different RNAseq *G. boninense* libraries, namely the monokaryon, dikaryon and the mating junction of two compatible monokaryons, were generated and analyzed [69]. These datasets were deposited and are publicly available in the NCBI database with accession number PRJNA269646. Several mating factors were up-regulated in the mating junction library, including the pheromone receptor, *STE3* [70], which is involved in the mating signaling pathway of other fungi [71], thus confirming their function in the formation of the dikaryon in *G. boninense*.

To further identify the pathogenicity factors produced by this fungus during infection, RNAseq analyses of the fungus were carried out on the infected plant host. The RNAseq data is available in the NCBI database with the accession number PRJNA514399 and is the first reported data to uncover the molecular mechanisms and pathways during *G. boninense* infection toward the oil palm *in planta*. However, external environmental factors such as temperature, osmotic stress and moisture have affected the expression levels of several pathogenicity genes [72]. Several genes including CAZymes, which belongs to six families of auxiliary activities (AA) enzymes—such as five multicopper oxidases (AA1_1), four glucose-methanol-choline (GMC) oxidoreductases (AA3) and a copy of peroxidase (AA2), copper radical oxidase (AA5), and benzoquinone reductase (AA6)—were up-regulated in the fungal–plant interaction [49]. Besides, a copper-dependent lytic polysaccharide monooxygenase (LPMO) which belongs to the AA9 (formerly GH61) family was also up-regulated during its interaction with the plant host [49] (Table 2). The oil palm tree expressed several genes as its defense, such as the pathogenesis-related protein 1-like (*EgPR-1*), expansin-B18-like (*EgEXPB18*) and chitinases (*EgCht*), which were significantly up-regulated during the early stage of infection (3 and 7 days post-inoculation). Besides, some genes were down-regulated during the infection by *G. boninense*, such as GDSL esterase/lipases 5 (*EgGLIP5*) and monogalactosyldiacylglycerol synthase 1 (*EgMGD1*) [43], which has been simplified in Table 2.

Phytopathogenic fungi, including *G. boninense*, secrete several non-catalytic proteins, including cerato-platanin proteins (CPPs), which play essential roles in the interactions between fungi and hosts. Many CPPs are available in plant pathogenic fungi, and these proteins serve as virulence factors in the interactions of fungi with plants [73]. Our preliminary analyses have identified 22 CPPs in the genome of *G. boninense* G3 (unpublished data). Generally, CPP encoding genes’ expression was higher during the early phase of infection [68]; however, a previous study has shown that *G. boninense* CPP is down-regulated during the infection [49]. We hypothesize that the infection was at the late phase because the sample was collected 30 days after inoculation. Thus, the fungus did not require CPPs to be expressed, as the host immune system had been activated.

RNAseq data sequences of infected oil palm tissues from three different conditions, including the healthy section of infected oil palm (IPHT), the near-rot section of infected oil palm (IPIT) and the cross-section of a healthy tissue section of healthy oil palm tree (HPHT), have been generated [74]. These data will undoubtedly lead to a better understanding of the genes responsible for, and implicated in, *G. boninense* infection toward its host. The raw RNAseq reads are available in the BioProject (NCBI) with the accession number PRJNA530030.

Several genes encoding CWDEs, especially transcripts involved in the lignin degradation process, including the laccase genes, were up-regulated in a carbon-rich culture incorporating oil palm sawdust [48]. Laccase has diverse biological functions, and several studies have shown that fungal laccases also play a role in pathogenicity. *G. boninense*‘s genome possesses 33 laccase encoding genes. However, the expression levels of these laccase genes were unique depending on the aggressiveness of the *G. boninense* strain; high virulence (I13), moderate virulence (NJ3) and low virulence (G13) with 11, 7 and 5 laccase genes, respectively, were up-regulated [48]. It was postulated that the differentially expressed laccase transcripts between isolates were related to the capability of the strain to degrade the stem. In addition, another study has shown that two cyclophilin genes (GbCYP203 and GbCYP205) were up-regulated during in vitro infection toward the oil palm and were postulated to be involved in *G. boninense* pathogenicity [75]. Furthermore, another gene postulated to play a role in the pathogenicity of *G. boninense*, known as necrosis and ethylene-inducing 2 protein (GbNEP), was studied. The gene encoding GbNEP was cloned and expressed recombinantly in a bacterial system. The infection assay of the recombinant protein (in vitro) revealed that the rGbNEP could induce necrosis in tobacco and tomato. However, the rGbNEP was unable to induce the same symptoms in oil palm leaves and root tissues [76].

Studies on the global gene expression analysis of the host response after the inoculation with *G. boninense* have shown that the plant host expresses several genes that are required in the biosynthesis of phytohormones such as ethylene, methyl jasmonate (MeJA) and methyl salicylate (MeSA). Furthermore, several antioxidants, such as L-ascorbate and myoinositol, were also highly expressed, whereas many genes required for photosynthesis were down-regulated during infection by this fungus [53]. The oil palm also produces PR proteins such as protease inhibitors, chitinases and secondary metabolites that possess fungicidal properties against *G. boninense* as its defense response to the fungal pathogen [43,52]. All data generated from these different studies could be used to determine the genes responsible for infecting the oil palm tree (Table 3).

## 5. Detection and Control Strategies

To date, no disease management method has been effective [10] in preventing the continuing spread of *Ganoderma* disease. The primary mode of infection occurs in the soil, thus making detection difficult [78]. Current detection methods rely on observing visible symptoms exhibited by infected trees, such as a yellowing and orange discoloration of the leaves, withering, crown flattening and unopened spear leaves [79]. However, this detection method sometimes results in misdiagnosis because these symptoms could also be caused by other factors, such as malnutrition and drought. The appearance of *G. boninense* fruiting bodies on the infected trees indicates that the disease is already at the late stage of infection [7]. In advanced cases, the oil palm stem might rupture [42], thus restricting the absorption of water and nutrients from the roots to the leaves, leading to chlorosis [13].

A typical early detection method for BSR involves the drilling of suspected infected plant material from the palm tree. The plant sample is then cultivated on selective media to detect the presence of *Ganoderma* [80]. This method is time-consuming and inaccurate, and the disease is already well-established by the time results are generated. Molecular detection methods began to be developed after 2000 due to the advancement of techniques during that time. A PCR-based detection was developed, involving a combination of primers used to amplify specific markers for *Ganoderma* detection [81]. A detection method utilizing an immuno-based assay by an enzyme-linked immunosorbent assay (ELISA) using monoclonal antibodies was developed [82]. The immunosorbent assay has the advantage of being more straightforward and faster compared to PCR; however, the results were varied and inconsistent [83]. In addition, these assays produced false positives and cross-reactivity with other fungi commonly found in oil palm plantations [82,84].

Currently, research has been focused on developing new detection methods that are fast and highly specific. Loop-mediated isothermal amplification (LAMP)-based detection was developed because it is highly sensitive as compared to PCR-based techniques, where it only requires a 0.002 ng/μL DNA template compared to the 0.02 ng/μL needed for PCR-based detection methods [85]. In addition, the LAMP primer pair designed to amplify *bug1A* showed the potential to be used in detection because it can specifically identify and differentiate pathogenic *Ganoderma* species (such as *G. boninense*, *G. zonatum* and *G. miniatocinctum*) from other non-pathogenic *Ganoderma* [85]. Moreover, this LAMP-based detection technique is superior in detecting pathogenic *Ganoderma* species as compared to ELISA, which results in cross-reactivity and false-positive outcomes due to the unsuccessful blocking between the antigen and the *G. boninense* polyclonal antibody. Another advantage of using LAMP is its simpler sample preparation, minimizing cross-contamination and generating results within 30 and 60 min [86]. Furthermore, the researcher can alter the reaction mixture according to their preference, and the experiments can be visualized on-site by a portable machine.

Furthermore, several groups have focused on developing a new detection method using electrochemical sensors with different types of particles, including gold nanoparticles (AuNPs) and carbon nanotubes (CNTs) [87,88]. Recently, a combination of polymer pen lithography (PPL) and DNA–gold nanoparticle (DNA–AuNP) conjugates were developed for *G. boninense* [89]. This technique provides better precision in detecting *G. boninense* in plant samples, including the unamplified genomic DNA of this fungus. However, these electrochemical sensing methods are costly and time-consuming and can only detect BSR at the later stage of infection. Therefore, it is vital to develop faster and cheaper methods for detecting this fungus, especially at the early stages of infection.

Various methods have been used to control BSR, including soil mounding and surgical removal of dead plant tissue, together with the basidiocarps and chemical treatments by injecting fungicides into the tree [13,79]. Some plantations, however, still practice the burning of infected materials, creating environmental issues in the region.

Several fungicidal chemicals have been tested for *Ganoderma*, and one of the most widely tested chemicals in the field is hexaconazole [40,90]. Hexaconazole is able to reduce the risk of *Ganoderma* infection in healthy mature palm trees [34]; however, field tests have shown that the fungicide residues are moderately present in leaf samples up to 70 days after the treatment [91]. Another chemical that shows potential in *Ganoderma* treatment is a microgranular fumigant known as dazomet, which emits a toxic gas known as methyl isothiocyanate (MITC) when it interacts with water [92]. Dazomet treatment (1000 g) can prevent the growth of a *Ganoderma* inoculum up to 90% in infected stumps [93]. A recent study identified pyraclostrobin, which could be used as a fungicidal agent against *G. boninense*. Pyraclostrobin has shown dual functions: suppressing *G. boninense* while concomitantly improving plant growth. Pyraclostrobin also induces the host-defense-related gene, β-1,3-glucanase [94]. However, further investigation is still required to determine the effect of the continuous usage of this fungicide on the biodiversity of the soil microbiome, as well as its effect on the environment.

With numerous unsuccessful attempts to reduce the disease and the concerning effect of fungicides on the environment, the current focus of research has shifted toward identifying potential biological control agents against the fungus. One of these focuses is the identification of microbial antagonists to the phytopathogen. Although several potential microbial biological control agents have been identified, including bacteria [12,95], actinomycetes [95,96], endophytes [97], fungi [98] and seaweed [99], these biological control methods do not act universally toward *G. boninense* (Table 4). An efficient microbial biological control agent with a high percentage inhibition of radial growth (PIRG) values toward one strain of *G. boninense* did not exhibit similar high PIRG values toward a different dikaryotic strain of the same fungus. This is believed to be compounded by the tetrapolar *G. boninense* mating system, which renders it highly capable of diversifying its genetic makeup, leading to a variation in genome composition [100]. An advantage of bio-control agents is their ability to evolve and adapt along with the phytopathogen, often showing primacy over them. At the same time, it must be noted that their ability to adapt determines not only the success of the control of the target pests, but also the possibility (and extent) of non-target effects. Thus, follow-up studies or long-term monitoring needs to be considered to properly assess the effect of bio-control agents in field conditions outside of controlled laboratory settings. Furthermore, questions have also been raised regarding the execution of this method, especially regarding the cost and reliability of biological agents in alternative disease control strategies [10,100,101].

Biological control agents often secrete specific substances, otherwise known as secondary metabolites, responsible for their fungistatic and/or fungicidal effects on fungal pathogens [103]. Currently, a few compounds isolated from different microbial species were able to inhibit the growth of *G. boninense* in vitro. For example, *Trichoderma virens* produce specific compounds such as phenylethyl alcohol (PEA), 3,4-dimethylpent-2-en-1-ol and dodecanoic acid [98,104], whereas a mixture of several *Bacillus* spp. and *Trichoderma* spp. produced pyrene-1,6-dione, N-acetyl-leu-leu-tyr-amide and 12-deoxyaklanonic acid [105]. A powdered mixture containing bio-control agents *Streptomyces* sp., such as *Streptomyces hygroscopicus*, and known antifungal producer *Streptomyces noursei* showed a strong inhibition (PIRG = 100) against *G. boninense* in vitro [102]. Furthermore, this formulation also reduced the disease incidence in oil palm seedlings to up to 73% after 6 months of *G. boninense* inoculation [102]. *Streptomyces* sp. was known to produce several antifungal compounds which might inhibit the growth of *G. boninense,* such as ribostamycin, salinomycin and benzylmalic acid [106]. Besides that, another *Streptomyces* sp. known as *Streptomyces palmae* CMU-AB204^T^ also show the potential to be used as a bio-control agent against *G. boninense*. The treatment of this microbe against *G. boninense* showed a reduced percentage of disease severity (DS) of 81.6% and reduced the severity of foliar symptoms (SFS) to 3.7% when the free-spores were applied to the seedlings. Moreover, DS and SFS were also reduced to 75.8% and 4.5% when the spores were encapsulated with alginate beads [107]. This actinomycete produced several bioactive compounds, such as actinopyrone A, anguinomycin A and leptomycin A, which inhibited the growth of *G. boninense* [107,108]. These compounds have the potential to be commercialized; however, further studies need to be carried out before they are used in oil palm plantations.

Recently, a new fertilizer known as GanoCare^®^ was formulated from a combination of powdered empty fruit bunches (EFB) with a “beneficial element”, including a higher content of calcium (Ca), silicon (Si), zinc (Zn) and boron (B) [109]. While the application of this fertilizer significantly reduces the disease incidence by up to 70%, more in-depth studies are needed, specifically to uncover their effect on the biochemical responses of oil palms and the effect on oil production. Several studies suggested that these elements suppress several plant diseases and enhance the resistance to the BSR disease [110]. Besides, we believe that commercial GanoCare^®^ also comprised a consortium of microorganisms that are able to suppress the growth of *G. boninense*. GanoCare^®^ shows promise by improving oil palm growth and host resistance against *Ganoderma* infection [109]. Further molecular analysis is still needed, however, to uncover how GanoCare^®^ boosts the defense-related mechanisms of the plant host against the fungus.

## 6. Future Directions for R&D

Current studies have shown promising results toward determining the pathogenicity factors of this fungus using available molecular biology techniques. Next-generation sequencing could be the best option in elucidating the determinants of pathogenicity in this fungus. Although transcriptomic datasets between this fungus and plant hosts are publicly available, the implications gleaned from these datasets are not conclusive due to environmental factors. Thus, the most promising way forward in determining the pathogenicity factors is by infecting the oil palm tissue culture with *G. boninense* in a controlled environment to minimize external environmental factors. Recently, BSR incidents were predicted to increase due to climate change in 2100, especially in Malaysia and Sumatra [5,14]. The prediction is based on the fungus’s higher virulence, which could reduce the oil palm resistance to the disease due to the unsuitable climate for oil palm. This situation will increase the risk of the unsustainability of oil palm production for the world’s two largest oil palm producers.

Moreover, the draft genomes available are as yet incomplete assemblies, which poses a significant problem for accurate gene characterization. The difference in the number of genes generated is perhaps due to the incomplete or fragmented nature of the next generation sequencing (NGS) datasets. Thus, an improved genome assembly, perhaps by utilizing a hybrid genome assembly approach, is needed to accelerate molecular studies of this phytopathogen.

One of the fundamental issues in solving the enigma of this fungus is how the monokaryon selects a compatible partner to form the infective dikaryon. Transcriptome data proved useful in identifying the candidate factors, such as the *Ste3* gene, that are essential in activating the mating signaling pathway [70]. An *Agrobacterium*-mediated transformation has been successfully developed [37]. This technique could be utilized in functional genomics studies; however, the technique is not compatible with the *G. boninense* mycelia. Thus, promising genome editing based techniques such as the CRISPR/Cas9 system, conventional homologous recombination or transformation methods may serve as more promising tools to advance knowledge on *G. boninense*. Developing such methods will be vital for functional genomics studies of *G. boninense*, especially in identifying the virulence factors during the infection [34].

The most studied *Ganoderma* species is currently *G. lucidum*, due to its medicinal properties. Some studies have identified that *G. boninense* also exhibits antibacterial activity against few nosocomial-infection-related bacterial pathogens, including *Escherichia coli*, *Klebsiella pneumonia*, *Pseudomonas aeruginosa*, *Salmonella enterica*, *Staphylococcus aureus* and *Streptococcus pyogenes* [111]. A further study has identified two compounds, ergosterol and ganoboninketal, which possess a potent antibacterial activity against *S. aureus* and *S. pyogenes* [112]. However, clinical studies mainly on the effect of the antibacterial compounds still need to be carried out to determine the suitability of these compounds produced by *G. boninense* as drug candidates.

A recent study has identified four *Ganoderma* resistance loci in oil palm, where two of them were involved in regulating the incidence of the first *Ganoderma* symptoms, while the other two were involved in the death of palm trees [113]. Thus, this information is vital in developing a breeding program to produce new oil palm (*Elaeis guineensis* Jacq.) varieties that are more resistant to the fungal pathogen.

## 7. Conclusions

Studies have shown the mechanism of infections governed by *G. boninense* which stealthily penetrate and colonize the host cell. Moreover, with the availability of NGS technologies, few genes related to the pathogenicity of this fungus have been identified. However, there are no disease management strategies that can efficiently stop the *Ganoderma* disease’s continuing spread. Future research should focus on the variability of the genetic content between the fungal strains at the molecular level, as this factor has contributed a variable tolerance to the current disease treatments. Besides, developing a suitable method for functional genomic analysis would complement and gear up the development of new approaches to cure the infected palms or control the spread of this fungus, aiming to maintain sustainable oil palm production.

## Figures and Tables

**Table 1:** Current whole genomic and mitochondria data of *G. boninense* available in the public database.

Strain	Source of Isolate	Sample Type	Genome Size	Accession	Reference
PER71	Peninsular Malaysia, Malaysia	Genomic DNA	-	PRJNA182005	Broad Institute
NJ3	North Sumatra, Indonesia	Genomic DNA	65.03 Mb	PRJNA287769	[64]
G3	North Sumatera, Indonesia	Genomic DNA	79.24 Mb	PRJNA421251	[63]
FGV-M	Peninsular Malaysia, Malaysia	Genomic DNA	66.57 Mb	PRJNA503786	[66]
BRIUMSc	Borneo, Malaysia	Genomic DNA	52.28 Mb	PRJNA553124	[65]
G3	North Sumatera, Indonesia	Mitochondrial DNA	86,549 bp	PRJNA421251	[67]

**Table 2:** List of up-regulated and down-regulated genes between *G. boninense* and the plant host seedlings, *E. guineensis*.

Gene(s)	Up-Regulated Genes	Down-Regulated Genes
**Fungus (*G. boninense*)** [49]		
**Plant Cell Wall Degrading Enzymes (CWDEs)**		
multicopper oxidases (AA1_1)	/(5)	-
glucose-methanol-choline (GMC) oxidoreductases (AA3)	/(4)	-
peroxidase (AA2)	/(1)	-
copper radical oxidase (AA5)	/(1)	-
benzoquinone reductase (AA6)	/(1)	-
copper dependent lytic polysaccharide monooxygenase (LPMO) (AA9)	/(1)	-
xyloglucan hydrolases (GH16)	/(2)	-
carboxylesterase enzymes	/(2)	-
α-glucosidase (GH31)	/(1)	-
β-galactosidases (GH35)	/(2)	-
α-glucuronidases (GH15)	/(2)	-
β-glucuronidases (GH79)	/(2)	-
pectate lyases 3 (PL3)	/(2)	-
pectate lyases 8 (PL8)	/(1)	-
**Fungal Cell Wall Remodeling and/or Degrading Enzymes**		
chitin synthase (CHS)	/(1)	-
chitinase	/(1)	/(5)
endochitinase	/(1)	-
Beta-glucanase	-	/(2)
**Small Secreted Proteins**		
hydrophobins	-	/(4)
cerato platanins	-	/(3)
**Stress Response Proteins**		
thaumatin-like proteins	-	/(5)
**Protease**		
metalloproteases	/(5)	-
**Oil palm (*E. guineensis*) root tissues** [43]		
**Pathogenesis-Related (PR) Proteins**		
pathogenesis-related protein 1-like (*EgPR-1*)	/	-
peroxidases (*EgPER*)	/	-
germin-like proteins (*EgGLP*)	/	-
chitinases (*EgCht*)	/	-
**Secondary Cell Wall Biosynthetic Genes**		
cellulose synthase A catalytic subunits (*EgCESA*)	/	-
cellulose synthase-like proteins (*EgCSL*)	/	-
expansin-B18-like (*EgEXPB18*)	/	-
**Lipid Metabolism**		
GDSL esterase/lipases 5 (*EgGLIP5*)	-	/
monogalactosyldiacylglycerol synthase 1 (*EgMGD1*)	-	/
**Biosynthesis of Phytohormones**		
allene oxide cyclase 1, chloroplastic-like (*EgAOC1*)	-	/
12-oxophytodienoate reductase 1-like (*EgOPR1*)	-	/
1-aminocyclopropane-1-carboxylate oxidase-like (*EgACO*)	-	/
L-ascorbate L-gulonolactone oxidase-like (*EgGULO*)	-	/

(/) = Yes. (-) = No. Number of detected genes in parentheses.

**Table 3:** Current *G. boninense*-related transcriptomic data are available in the public database.

Transcriptome Data	Sample	Accession	Reference
*G. boninense*	Monokaryon Dikaryon Mating Junction	PRJNA269646	[69]
*G. boninense* at	axenic culture pathogen–oil palm interaction	PRJNA514399	[72]
Oil palm root	infected with *G. boninense* infected with *G. boninense + Trichoderma harzianum*	PRJEB7252	[52] [77]
Oil palm	infected by *G. boninense*	PRJNA530030	[74]
Oil palm leaf	infected with *G. boninense*	PRJEB17971	[53]
Oil palm	early interaction with *G. boninense*	PRJEB27915	[43]

**Table 4:** In vitro test of bio-control agents against *G. boninense*.

Bio-Control Agent	Dual Culture		Other Test		Reference
	PIRG Value (%)	Test	PIRG Value (%)	Test	
*Pseudomonas aeruginosa*	70.0	Dual culture	80.0	Culture filtrate	[12]
*Burkholderia cepacia*	55.5	Dual culture	65.0	Culture filtrate	[12]
*Streptomyces hygroscopicus*	50.0–80.0	Dual culture	100	Powder formulation	[96,102]
*Streptomyces ahygroscopicus*	50.0–80.0	Dual culture	100	Powder formulation	[96,102]
*Aspergillus calidoustous* BTF07	49.5	Dual culture	-	-	[97]
*Trichoderma asperellum* T2	47.5	Dual culture	-	-	[97]
*Trichoderma virens* 159C	-	-	44.3	Crude extract	[98]
*Sargassum oligocystum*	38.64	Dual culture	42.5	Hexane extract	[99]

## Data Availability

Not applicable.
